# Longitudinal Blood Epigenetic Aging, DNA Methylation-Predicted Protein, and Estimated Leukocyte Proportion Trends in Two Astronauts from the Axiom Space Mission 1: An Exploratory Analysis

**DOI:** 10.3390/genes17050564

**Published:** 2026-05-14

**Authors:** Jamaji C. Nwanaji-Enwerem, Dennis Khodasevich, Jermaine Blakley, Jonathan M. Galazka, Andres Cardenas

**Affiliations:** 1 Department of Emergency Medicine, Center for Health Justice, Center of Excellence in Environmental Toxicology, Perelman School of Medicine, University of Pennsylvania, Ground Ravdin, HUP, 3400 Spruce Street, Philadelphia, PA 19104, USA; 2 Department of Epidemiology and Population Health, Stanford University, Palo Alto, CA 94305, USA; 3 College of Medicine, Howard University, Washington, DC 20059, USA; 4 Space Biosciences Division, NASA Ames Research Center, Moffett Field, CA 94035, USA; 5 Department of Pediatrics, Stanford University, Stanford, CA 94305, USA

**Keywords:** DNA methylation, proteomic signatures, microgravity, immune cell dynamics

## Abstract

**Background/Objectives:** Spaceflight presents a combination of physical and psychosocial stressors that may impact biological aging and health. Understanding how spaceflight influences molecular aging processes is essential as commercial and professional space travel continue to expand. **Methods:** We analyzed publicly available DNA methylation data to evaluate longitudinal changes in 10 epigenetic aging biomarkers, 6 leukocyte proportion estimates, and 109 DNA methylation-derived protein scores in two astronauts participating in Axiom Space’s AX1 17-day low Earth orbit mission. We calculated mean values for all biomarkers across three timepoints: two weeks before spaceflight (T0), 24 h after spaceflight (T1), and three months after spaceflight (T2). Using the mean values, we next calculated the fold change from baseline for all biomarkers. Because the sample size precluded statistical testing, we identified the top 5% of absolute fold changes to highlight the largest shifts across candidate biomarkers. **Results:** Across epigenetic clocks, MiAge showed the greatest T0–T1 decrease (−4.26-fold), and DNAmFitAge showed the greatest T0–T2 increase (2.47-fold). NK cells exhibited the largest T0–T1 change, decreasing by 49% (−0.49-fold). B cells exhibited the largest T0–T2 change, decreasing by 11% (−0.11-fold). Proteins meeting a predefined top 5% fold change from baseline criterion at both T1 and T2, included BMP1, CLEC11A, CXCL11, FAP, and LTF. Enrichment analysis indicated involvement of serine-type endopeptidase activity, molecular function activator activity, and cell aggregation pathways. **Conclusions:** These findings suggest that spaceflight influences methylation-derived biomarkers of aging and immunity even in short-duration missions. These results, though exploratory, contribute to emerging efforts to characterize molecular resilience and vulnerability in human spaceflight.

## 1. Introduction

Physical and psychosocial environmental exposures are well-known contributors to aging-related conditions, including cancer, immune dysfunction, musculoskeletal decline, and slowed cognitive processing [1,2,3,4,5,6]. Furthermore, these exposures often interact synergistically rather than independently, and occupational contexts can further shape the nature and intensity of exposures. Astronauts represent a uniquely informative group in this regard because spaceflight combines multiple extreme environmental stressors, including microgravity, cosmic ionizing radiation, circadian disruption, confinement, and social isolation [7,8]. These spaceflight exposures are believed to contribute to increased risks of several disease processes in astronauts, including melanoma skin cancer, cardiovascular events, cataracts and other eye diseases, and decreases in bone mineral density [9,10,11,12]. As opportunities for commercial space travel continue to expand and the number of people exposed to these conditions is likely to grow, it is increasingly important to understand how spaceflight contributes to human aging and overall health.

There is growing interest in using molecular biomarkers to characterize how spaceflight influences biological aging and overall health. DNA methylation–based epigenetic clocks (epigenetic age) have emerged as robust indicators of biological age and are sensitive to a wide range of environmental exposures [13,14,15]. These measures capture cumulative molecular changes associated with chronological age, disease risk, and mortality [16,17,18]. Despite this promise, research examining relationships between epigenetic aging and spaceflight remains limited. In the Mars-500 520-day ground simulation experiment involving six astronauts, investigators reported significant decreases in PhenoAge, a measure associated with morbidity, and GrimAge, a measure associated with mortality, during the mission [19]. Only the PhenoAge decline remained significant in the post-mission period. The study also noted decreases in estimated leukocyte subsets including plasmablasts, NK cells, CD8 T cells, and CD4 naïve T cells, whereas B cells, CD4 T cells, and monocytes increased over the course of the mission. However, only changes in NK cells were statistically significant after mission completion [19]. In contrast, a recent study of four astronauts participating in the 9-day Axiom-2 mission to the International Space Station found that participants showed epigenetic age acceleration while in flight, followed by deceleration within 24 h of returning to Earth. The authors further implicated regulatory T cells, naïve CD T cells and neutrophils as contributors to these patterns [20].

To build on this emerging body of knowledge, the present exploratory study examines publicly available DNA methylation data from two astronauts who participated in Axiom Space’s AX1 mission, a 17-day low Earth orbit flight. We evaluate longitudinal relationships between mission duration and both epigenetic aging measures and estimated leukocyte proportions. Recognizing that proteomic alterations may reflect downstream functional consequences of epigenetic regulation, immune modulation, metabolic adaptation, and stress response pathways, we also assess longitudinal changes in methylation-based protein scores that approximate circulating levels of 109 plasma proteins [21]. Of note, these methylation-derived protein scores, while imperfect proxies, were developed using directly measured protein levels and demonstrate significant correlation with their corresponding circulating proteins [21]. We have also previously applied this approach in an independent cohort, where surrogate-based findings were consistent with results from corresponding proteomic analyses [22]. Because our analysis focuses on a mission longer than the previously studied 9-day Axiom mission but shorter than the 520-day Mars-500 simulation, we hypothesize that patterns in epigenetic aging biomarkers and leukocyte profiles may share features with both prior studies yet not fully mirror either. By additionally incorporating predicted circulating protein profiles, we aim to provide a more comprehensive framework for characterizing biological aging trajectories and to highlight potential mechanistic pathways linking spaceflight exposure to physiological resilience or vulnerability.

## 2. Methods

### 2.1. Study Sample

Our analysis used publicly available DNA methylation data downloaded from the NCBI Genome Expression Omnibus (GEO) from two astronauts who participated in Axiom Space’s privately funded and operated mission to the International Space Station (GSE276323). The 17-day low Earth orbit mission launched on 8 April 2022, with a fully privately operated and funded crew of four individuals. Only data for two of the four participants was publicly available. Limited phenotypic data on these two participants (OSD-943) was available and downloaded from the NASA Open Science Data Repository (OSDR). The phenotypic data included participant sex, a unique de-identified identifier, and the timepoint of each sample. Both participants were male and provided blood samples at three time points: two weeks before spaceflight (T0), 24 h after spaceflight (T1), and three months after spaceflight (T2).

### 2.2. DNA Methylation Analysis and Calculating Epigenetic Age, Leukocyte Proportions, and Estimated Protein Levels

DNA methylation was measured using the Illumina Infinium MethylationEPIC BeadChip (Illumina, San Diego, CA, USA). DNA methylation IDAT files were accessed from GEO using accession number GSE276323. IDATs were imported into R for processing using the recommended SeSAMe pipeline including masking probes of poor design, inferring channel for Infinium-I probes, non-linear dye bias correction, detection *p*-value masking using oob, and background subtraction using oob, and excluding sites missing in >2/6 samples [23]. We then used the *methscore* function from the *ENmix* package to calculate epigenetic age measurements and epigenetic scores for 109 circulating protein biomarkers [21,24]. *Methscore* imputes any required CpGs that are missing using reference data before calculating epigenetic age measurements and protein scores [24]. The degree of missingness of component CpG sites for each predictor is summarized in Table S1. Given that we had limited phenotypic data on the study participants and that chronological age may be an important confounder in these analyses, we first calculated the Horvath2 epigenetic aging measure which is regarded as one of the most accurate predictors of chronological age, and used this measure (at baseline) as a surrogate for chronological age in all subsequent analyses [25]. We calculated DNA methylation-estimated leukocyte proportions (granulocytes, monocytes, CD4T, CD8T, NK, and B cells) using the *ewastools* package [26,27].

We examined all DNA methylation–based biomarkers of biological aging, fitness or frailty, cell division history, and telomere length available from the *methscore* output. Where principal component–based versions of biomarkers were available, we used those estimates because they reduce technical noise. These biomarkers included ten established epigenetic clocks: PC PhenoAge, PC GrimAge, DunedinPACE, Frailty, Zhang10CpG Mortality, bAge, DNAmFitAge, EpiTOC2, MiAge, and PC DNAmTL. PC PhenoAge and PC GrimAge represent principal component–derived versions of the PhenoAge and GrimAge clocks, which are predictors of morbidity and mortality risk, respectively [16,28,29]. DunedinPACE reflects the pace of aging, measured as the number of biological years accrued per chronological year [30]. The Frailty clock represents a methylation-based frailty index [31]. The Zhang10CpG score is a mortality risk estimator based on 10 CpG sites [32]. The bAge clock provides an estimate of biological age [33]. DNAmFitAge incorporates molecular correlates of physical fitness [34]. EpiTOC2 and MiAge index mitotic history and cumulative cell divisions [35,36]. PC DNAmTL reflects methylation-estimated telomere length [29,37].

### 2.3. Statistical Analysis

We conducted a longitudinal analysis of repeated biological measurements across three time points (T0, T1, and T2). For each time point, we used estimated leukocyte proportions as well as *methscore* produced epigenetic clock and predicted protein level residual values that accounted for chronological age via Horvath2 age as a surrogate. We calculated means of leukocyte proportion, epigenetic clock, and predicted protein residuals across the two study participants. Because the study included only two participants, statistical tests for comparing means and determining significance were not applied. Instead, we evaluated the magnitude of change by calculating the mean relative fold change from baseline (Δ = (TX − T0)/|T0|) for each leukocyte proportion, epigenetic clock, and protein measure. We calculated the fold change at both T1 and T2 compared with baseline (T0). To assess the magnitude of change and to highlight the most substantial shifts over time, we identified biomarkers whose fold changes fell within the top 5% of the absolute distribution of all observed fold changes. This percentile-based threshold is inherently arbitrary and was selected as a transparent, convention-driven heuristic that is conceptually similar to widely used statistical cutoffs such as *p* < 0.05 and was intended to prioritize the largest observed changes for focused discussion in an exploratory, underpowered dataset. Importantly, the threshold was used solely as an interpretive aid, and all biomarkers and trends are reported to allow readers to draw independent conclusions.

Lastly, we used the g:Profiler platform to perform pathway enrichment analyses of our highlighted proteins [38]. Our analysis included 109 DNA methylation–based protein surrogates, so we first evaluated all surrogates to establish a background set of enriched pathways. We then performed a separate enrichment analysis on the subset of proteins that met our top 5% threshold criteria to identify pathways enriched beyond those observed in the background set. All statistical analyses were performed using R version 4.4.1 (R Core Team, Vienna, Austria).

## 3. Results

Participants had baseline Horvath2 ages of 46.4 and 58.1 years. We observed mean increases in the fold change from T0 to T1 for B cells, CD4 cells, and granulocytes. In contrast, CD8 cells, monocytes, and NK cells showed decreases from baseline to T1. NK cells exhibited the largest absolute baseline fold change during this interval, decreasing by 49% (−0.49-fold) from T0 to T1. This shift was not sustained when comparing NK cell means from T0 to T2, which showed an increase of 2% (0.02-fold). From T0 to T2, CD4 cells, CD8 cells, and NK cells increased relative to baseline, whereas B cells, granulocytes, and monocytes decreased. B cells demonstrated the largest absolute baseline fold change from T0 to T2, decreasing by 11% (−0.11-fold) (Table 1).

Of the ten epigenetic clock measures assessed, the means of four decreased from baseline to T1 (DunedinPACE, EpiTOC2, MiAge, and PC DNAmTL), while the remaining measures increased during the same interval. Only DunedinPACE, EpiTOC2, and PC DNAmTL remained lower than baseline at T2. MiAge showed the largest absolute fold change from T0 to T1, decreasing by 426% (−4.26-fold). DNAmFitAge demonstrated the greatest absolute fold change from T0 to T2, increasing by 247% (2.47-fold) (Table 2).

**Figure 1:**
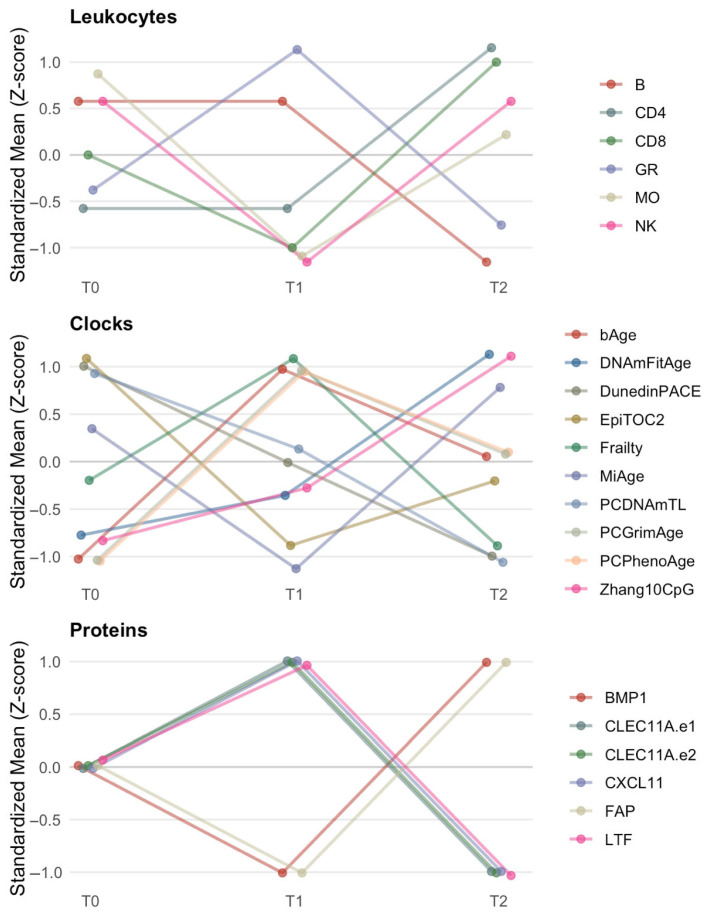
Timepoint Trends. This figure displays within-biomarker standardized (z-score) trajectories for leukocyte proportions, epigenetic clocks, and DNA methylation–based protein surrogates across time points T0–T2. Using within-biomarker z-score standardization allows us to display relative trends across heterogenous scales. Lines are plotted with slight horizontal staggering and semi-transparent styling to enhance visibility of overlapping markers.

Using our arbitrary, but thoughtful and predefined criterion that identified proteins within the top 5% of absolute fold changes at both T1 and T2, six of the 109 predicted proteins met this threshold. BMP1 and FAP showed the largest bidirectional shifts, first decreasing from baseline to T1 by 39-fold and 49-fold, respectively, and then increasing from baseline to T2 by 36-fold and 46-fold, respectively. CLEC11A.e1, CLEC11A.e2, CXCL11, and LTF showed increased mean levels from baseline to T1, followed by decreases relative to baseline at T2 (Table 3). Results for all remaining proteins are presented in Table S2. The proteins meeting the top 5% criterion showed statistically significant enrichment for serine-type endopeptidase activity, molecular function activator activity, and cell aggregation pathways (Table 4). Of these, only serine-type endopeptidase activity was also enriched in the total 109 surrogate protein list. All leukocyte, clock, and protein trends are depicted in Figure 1.

## 4. Discussion

In this exploratory longitudinal analysis of two male astronauts, we examined changes in estimated leukocyte composition, epigenetic aging measures, and DNA methylation-based predicted protein levels to better understand how these biological features shift in response to a space mission. Although the small sample size limited the use of traditional statistical testing, our fold change framework provided us with a means to identify the most pronounced differences across the three time points: two weeks before spaceflight (T0), 24 h after return (T1), and three months after return (T2). Several preliminary patterns emerged. For leukocytes, NK cells showed the largest early shift with a 49% decrease from T0 to T1, while B cells demonstrated the greatest change from T0 to T2. Among epigenetic aging measures, MiAge exhibited the largest decrease from T0 to T1, and DNAmFitAge showed the largest increase by T2. For predicted proteins, six met our top 5% threshold at both T1 and T2, including BMP1 and FAP, which showed particularly large bidirectional changes across the mission period. Together, these findings suggest that space travel influences methylation-derived biomarkers, even in a small, short-duration study context. They may also offer complementary insights into existing work as well as to larger and better powered studies in this area.

From the Mars-500 mission, one might hypothesize that most epigenetic age measures would show deceleration following a space mission [19]. However, the Axiom-2 study suggests a different pattern, in which epigenetic age acceleration may occur first, followed by a later period of deceleration after returning to Earth [20]. Although we cannot speak to statistical significance, we observed several markers consistent with reduced aging shortly after the mission, reflected by lower mean values of DunedinPACE, EpiTOC2, and MiAge at the T1 time point. DunedinPACE quantifies the pace of aging [30]; EpiTOC2 and MiAge reflect mitotic history and stem-cell division rates [35,36]. Of these measures, the MiAge change was the largest in magnitude. However, only the EpiTOC2 and DunedinPACE trends persisted three months after the mission. In fact, some biomarkers demonstrated a reversal in direction between the 24 h post-mission and three-month post-mission measurements when compared with pre-mission levels. This pattern of reversal is not surprising. Prior work, including the NASA Twins Study, documented apparent age reversal during spaceflight through lengthening of biomarkers such as telomeres, only for those same markers to show accelerated aging later [39]. One possible explanation is that astronauts may cope effectively with the immediate stresses of spaceflight, but these short-term compensatory responses may carry physiological costs that become evident later in the post-mission period. Nonetheless, these assertions remain hypotheses and we cannot fully rule out that our results are due to noise or some other non-biological variation. Future studies with larger sample sizes will be essential for determining which of these relationships are statistically and biologically significant.

The top 5% trends in epigenetically estimated protein levels identified in our analysis included BMP1, CLEC11A, CXCL11, FAP, and LTF. BMP1 (Bone Morphogenetic Protein 1) is a secreted metalloprotease involved in extracellular matrix formation and tissue remodeling, and it plays an important role in cartilage and bone formation [40]. BMP1 has also been linked to bone mineral density and remodeling physiology in astronauts [41]. CLEC11A (C-type Lectin Domain Family 11 Member A), also known as stem cell growth factor, supports hematopoiesis and contributes to immune regulation and bone development [42,43]. CXCL11 (C-X-C motif chemokine ligand 11) is a chemokine that recruits immune cells to sites of inflammation and is often upregulated during interferon-related immune responses [44,45]. It is also overexpressed in several human cancers [46], and space radiation has been associated with upregulation of CXCL11 in experimental models [47]. FAP (Fibroblast Activation Protein) is a serine protease expressed in pathological conditions such as arthritis, fibrosis, and cancer [48]. LTF (lactotransferrin or lactoferrin) is an iron-binding protein involved in mucosal defense and innate immunity, and in some contexts it can act as a tumor suppressor [49,50]. Overall, these proteins participate in diverse biological processes, yet they converge on key themes linked to spaceflight, including bone remodeling, tissue repair, and immune defense.

Pathway analysis identified three enriched pathways in our predicted protein results. Although we found limited evidence linking molecular function activator activity to spaceflight, there was some support for connections involving serine-type endopeptidase activity and cell aggregation. Serine-type endopeptidases cleave peptide bonds and participate in a wide range of biological functions, including immune responses, blood clotting, and digestion [51]. Prior work has implicated these enzymes in the spaceflight context. In a study of six Russian cosmonauts completing 169 to 199 days on the International Space Station, urinary proteomic profiles collected before launch and on the first and seventh days after return to Earth showed that serine-type endopeptidases detected pre-flight were no longer present on the first day post-flight [52]. One of these related proteins, inter-alpha inhibitor H4, is an anti-inflammatory marker, and its absence may reflect altered inflammatory regulation following spaceflight [53]. This interpretation is consistent with our findings and with other studies reporting relationships between spaceflight and shifts in immune cell proportions [19,20]. In this study, we observed the greatest changes in NK cells 24 h after the mission and in B cells three months after the mission. NK cells are innate immune cells that provide rapid defense against virally infected and transformed cells, acting as an early frontline component of the immune response [54]. B cells are adaptive immune cells responsible for antibody production and long-term immunologic memory, and they play a key role in responding to new pathogens [55]. The decrease in both cell types is consistent with findings from the Mars-500 mission, which reported reductions in NK cell proportions and in plasmablasts, a specialized and highly active form of B cell, with increasing mission duration. Furthermore, although B cells initially increased during the Mars-500 mission, their levels were similarly decreased post-mission [19]. With respect to cell aggregation, one possible connection involves research showing that microgravity can alter the human microbiome, potentially by affecting the aggregation behavior of *Candida albicans*, a budding yeast species that is part of the microbiome [56]. When considered along with the role of serine-type endopeptidase activity, this result adds to the evidence that spaceflight may influence aspects of digestion and host–microbe interactions. Although we did not perform statistical testing, it was reassuring that we observed similar trends in monocytes, B cells, and NK cells as reported in previously published work in these astronauts [57].

Still, it is important to note that our pathway analysis using methylation-based protein surrogates differed from the pathways reported in a prior DNA methylation CpG and transcriptomic analysis of these same data. That analysis primarily identified pathways related to nervous system development, mitosis, protein metabolic processes, regulation of membrane potential, and apoptotic signaling [57]. These differences are likely due to our protein surrogates only representing a limited set of proteins without complete representation among common pathways [21]. Moreover, there may also be differences in the analytic approaches used. Although our DNA methylation-based surrogates are associated with their measured protein levels, they are imperfect [21]. Transcriptomic data reflect direct gene expression while methylation-based protein scores represent downstream functional surrogates rather than immediate transcriptional activity. Our findings, however, demonstrate some overlap with prior results in the area of metabolic processes. Consistent with what has been described in prior studies using methylation-based protein surrogates, we suggest that these markers can provide complementary insight into underlying biological pathways, but they should be interpreted alongside direct transcriptomic analyses whenever possible [22].

This study has several notable strengths. First, by leveraging publicly available DNA methylation data collected at three well-spaced time points before and after spaceflight, we were able to characterize changes in leukocyte composition, epigenetic aging measures, and predicted protein levels in a way that few prior studies have examined. Furthermore, the use of methylation-derived biomarkers allowed us to evaluate multiple layers of biological response simultaneously, including immune shifts, epigenetic aging trajectories, and proteomic signatures. Additionally, our fold change and top 5% approach also provided a practical framework for identifying the most pronounced changes in a setting where statistical power is limited. Finally, examining data from a 17-day mission allowed us to compare our findings to both shorter and longer spaceflight studies and to situate our results along a broader continuum of spaceflight exposure. Still, there are also important limitations that should be acknowledged. The study includes only two participants, which restricts statistical inference and limits generalizability. We also lacked demographic information for the two study participants, including chronological age and health history. As a result, we were unable to adjust for individual characteristics that may influence DNA methylation, immune composition, or protein profiles. Because chronological age was not available, we relied on a surrogate estimate, which may introduce additional uncertainty into the interpretation of aging-related biomarkers. Nevertheless, we employed the same Horvath2 substitution method in a previously published longitudinal analysis of 6 astronauts from the NASA Mars-500 mission [19]. Access to demographic and clinical information in future work will be important for placing observed molecular changes in the appropriate physiological context and for distinguishing spaceflight-related effects from individual variability. Furthermore, we relied on fold changes rather than formal statistical testing, which highlights major shifts but cannot determine significance or rule out chance findings. In this context, some large-magnitude fold changes, particularly for measures such as MiAge, may be influenced by small baseline values or sign changes across time points, which can mathematically inflate fold change estimates. Accordingly, these results should not be interpreted as literal or biologically proportional changes over the mission period, especially given the short duration of spaceflight and the small sample size. Instead, fold changes are intended to highlight the direction and relative extremeness of observed trends within an exploratory framework, rather than to imply specific quantitative effects on underlying biological processes. Moreover, DNA methylation estimates of leukocytes and proteins are indirect and may not capture all aspects of immune or proteomic activity, although they provide useful approximations and have been used for similar analyses in prior studies [21,22]. Despite these limitations, the study provides preliminary insight into molecular responses associated with spaceflight and can inform future work with larger cohorts.

## 5. Conclusions

In summary, this exploratory analysis provides preliminary insight into how short-duration spaceflight may influence leukocyte composition, epigenetic aging measures, and methylation-based predicted protein profiles. Even within a small two-person dataset, we observed changes across immune, aging, and proteomic biomarkers that suggest spaceflight can engage multiple biological pathways over relatively short timeframes. Although exploratory in nature given the limitations of our study sample, these findings add to the growing body of work suggesting that molecular responses to spaceflight are dynamic and multifaceted. As commercial space travel expands and a greater number of individuals are exposed to these environments, studies with larger cohorts, complete participant demographic/health data, repeated sampling, and integrated multi-omic designs will be critical for clarifying the biological impacts of spaceflight and for identifying factors that contribute to physiological resilience or vulnerability.

## Figures and Tables

**Table 1:** Longitudinal Trends in Estimated Leukocyte Proportions.

Leukocyte	T0 Mean	T1 Mean	T2 Mean	T1 Δ from T0	T2 Δ from T0
B	0.05	0.05	0.04	0.01	−0.11 *
CD4T	0.14	0.14	0.15	0.02	0.06
CD8T	0.08	0.07	0.09	−0.08	0.08
Granulocyte	0.65	0.69	0.64	0.08	−0.01
Monocyte	0.10	0.07	0.09	−0.27	−0.10
NK	0.05	0.02	0.05	−0.49 *	0.02

* Absolute value of Δ in top 5% of observations.

**Table 2:** Longitudinal Trends in Epigenetic Clocks.

Clock	T0 Mean	T1 Mean	T2 Mean	T1 Δ from T0	T2 Δ from T0
bAge	−0.38	0.36	0.02	1.96	1.04
DNAmFitAge	−0.61	−0.28	0.89	0.53	2.47 *
PC PhenoAge	−0.53	0.48	0.05	1.90	1.10
PC GrimAge	−0.39	0.36	0.03	1.92	1.08
DunedinPACE	0.02	−0.0003	−0.02	−1.01	−1.99
Frailty	−0.003	0.01	−0.01	4.02	−1.02
Zhang10CpG	−0.03	−0.01	0.04	0.82	2.18
EpiTOC2	17.37	−14.12	−3.25	−1.81	−1.19
MiAge	5.35	−17.42	12.07	−4.26 *	1.26
PC DNAmTL	0.01	0.002	−0.01	−0.64	−2.36

* Absolute value of Δ in top 5% of observations.

**Table 3:** Top 5% Absolute Value of %Δ Longitudinal Trends in DNA Methylation-Predicted Proteins.

Protein	T0 Mean	T1 Mean	T2 Mean	T1 Δ from T0	T2 Δ from T0
BMP1	0.00002	−0.001	0.001	−39.49	36.49
CLEC11A.e1	−0.0002	0.01	−0.01	31.60	−28.60
CLEC11A.e2	0.0002	0.01	−0.01	35.13	−38.13
CXCL11	−0.00004	0.002	−0.002	38.71	−35.71
FAP	0.00002	−0.001	0.001	−49.04	46.04
LTF	0.0001	0.001	−0.001	12.59	−15.59

**Table 4:** g:Profiler Enrichment Results.

GO Term	Term ID	Observed Protein Count	Matching Proteins	FDR *p* Value
Serine-type endopeptidase activity	GO:0004252	3	BMP1, FAP, LTF	<0.001
Molecular function activator activity	GO:0140677	4	BMP1, CLEC11A, CXCL11, LTF	<0.001
Cell aggregation	GO:0098743	2	BMP1, LTF	<0.001

## Data Availability

The datasets analyzed in the current study are available from the NCBI Genome Expression Omnibus (GEO) (GSE276323) and NASA Open Science Data Repository (OSDR) (OSD-943) websites.

## Supplementary Materials

The following supporting information can be downloaded at: https://www.mdpi.com/article/10.3390/genes17050564/s1, Table S1: Methscore CpG Summary; Table S2: Complete DNA Methylation-Predicted Protein Trends.
